# Ultra-Processed Foods and Metabolic Dysfunction: Mechanisms, Clinical Implications, and Public Health Perspectives. Position Paper of the Italian Association of Dietetics and Clinical Nutrition (ADI)

**DOI:** 10.1007/s13668-026-00790-0

**Published:** 2026-08-26

**Authors:** Giovanna Trinchese, Barbara Paolini, Fabiano Cimmino, Filippo Valoriani, Lucio Lucchin, Carmela Bagnato, Annarita Sabbatini, Massimiliano Petrelli, Pietro Losignore, Francesca Marino, Giulia Tavella, Odette Misa Sonia Hassan, Annalisa Maghetti, Maria Grazia Carbonelli, Sebastiano Banni, Luigi Barrea, Maria Pina Mollica

**Affiliations:** 1 Department of Biology, University of Naples Federico II, Naples, Italy; 2 Italian Association of Dietetics and Clinical Nutrition (ADI), Rome, Italy; 3 Azienda Ospedaliera Universitaria Senese, Siena, IT Italy; 4 Azienda Ospedaliero Universitaria di Modena-Policlinico, Modena, Italy; 5 Dietetics and Clinical Nutrition, Bolzano Health District, Bolzano, Italy; 6 UOSD Clinical Nutrition and Dietetic, Hospital Matera, Matera, Italy; 7 Dietetic and Clinical Nutrition Unit, IEO European Institute of Oncology IRCSS, Milan, Italy; 8 Endocrinology and Metabolic Disease Clinic, Azienda Ospedaliero-Universitaria delle Marche, Ancona, Italy; 9 Department of Neuroscience, Reproductive Science and Odontostomatology, University of Naples Federico II, Naples, Italy; 10 Diabetes Unit, “S. Camillo-Forlanini” Hospital, Rome, Italy; 11 Centro Specialistico di Dietetica e Nutrizione Clinica Matrik, Bologna, Italy; 12 Azienda Ospedaliera San Camillo Forlanini, Rome, Italy; 13 Department of Biomedical Sciences, Division of Physiology, University of Cagliari, Monserrato, Cagliari, Italy; 14 Dipartimento Di Psicologia E Scienze Della Salute, Centro Direzionale, Università Telematica Pegaso, Naples, Italy

**Keywords:** Dietary habits, Ultra-processed foods, Food quality, Inflammation, Homeostasis, Mitochondrial metabolism

## Abstract

**Purpose of Review:**

This position paper by the Italian Association of Dietetics and Clinical Nutrition (ADI) evaluates the impact of ultra-processed foods (UPFs) on metabolic health and chronic disease risk, with particular attention to the biological and behavioural mechanisms involved.

**Recent Findings:**

High UPFs consumption has been associated with obesity, insulin resistance, cardiovascular disease, metabolic dysfunction-associated steatotic liver disease (MASLD), cancer, neuroinflammation, and immune dysregulation. Proposed mechanisms include gut microbiota alterations, increased intestinal permeability, mitochondrial dysfunction, oxidative stress, impaired metabolic flexibility, and chronic low-grade inflammation. The hyperpalatable nature of UPFs may also impair appetite regulation and promote overconsumption, especially among children and adolescents. Additional concerns involve food additives, processing-derived contaminants, and endocrine disruptors from packaging materials.

**Summary:**

Current evidence identifies UPFs as a major modifiable risk factor for non-communicable chronic diseases. Reducing UPFs exposure should therefore represent a public health priority, supported by preventive nutritional strategies, improved food classification systems, and targeted educational and regulatory interventions.

**Graphical Abstract:**

Graphical Abstract. Ultra-Processed Food Consumption and metabolic dysfunction mechanisms. The high consumption of ultra-processed foods (UPFs) containing refined ingredients, additives, emulsifiers, and artificial flavouring agents lead to an increased intestinal permeability and dysbiosis. These alterations together with mitochondrial dysfunction and metabolic inflexibility lead to chronic low-grade inflammation. Furthermore, the high palatability of ultra-processed foods triggers hedonic eating mechanisms and encourage overconsumption. The interplay between microbiota-related altered metabolic signalling, increased adiposity and inflammatory processes contributes to an endocrine and metabolic disruption. These mechanisms may be particularly important during critical stages of development, such as childhood and adolescence, when dietary exposure can influence long-term metabolic programming and increase the risk of developing metabolic diseases later in life. Figure created with BioRender.com

**Figure:**
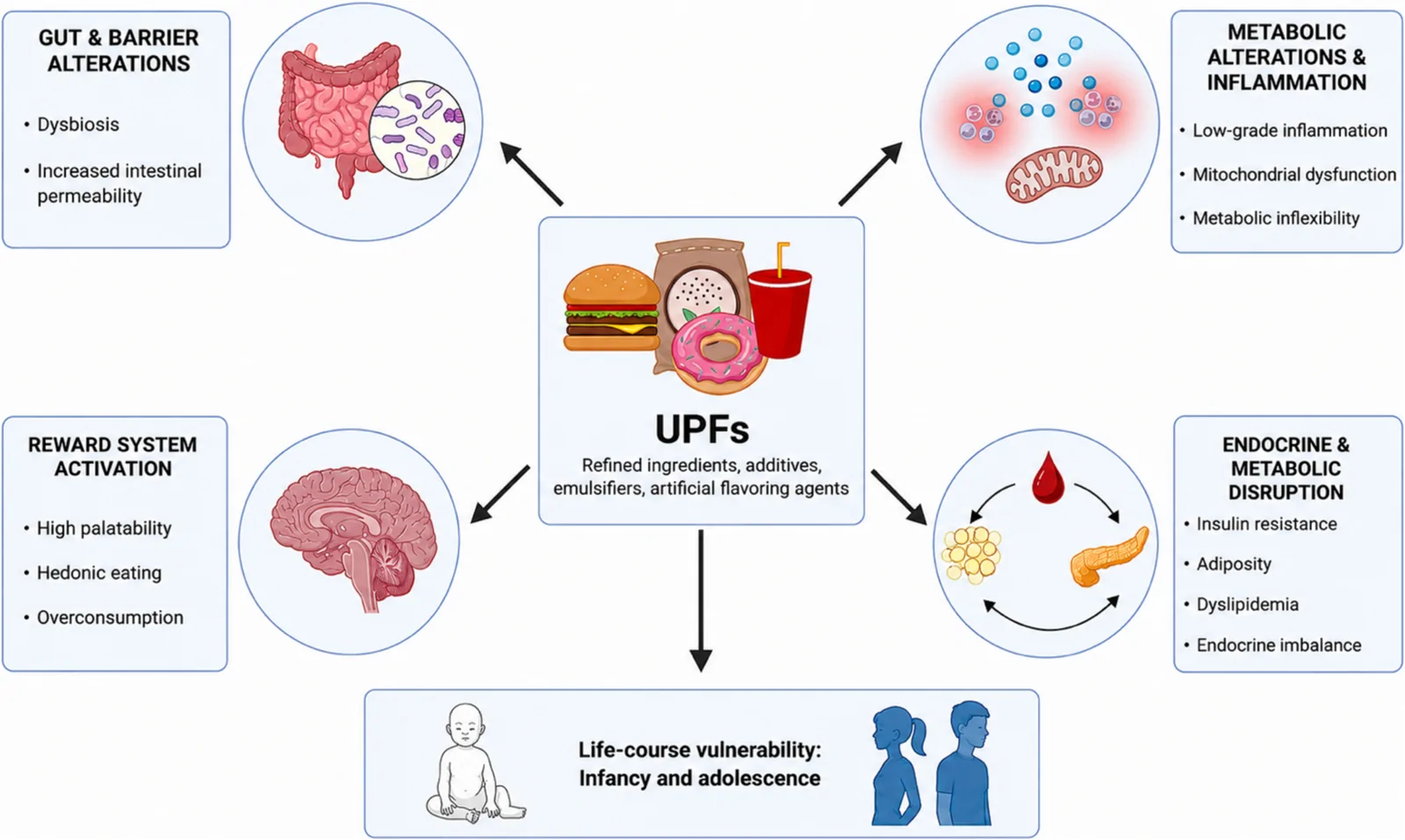

## Introduction

Food processing has been a part of human civilisation for millennia, playing a fundamental role in improving food safety, preserving seasonal produce, reducing waste and ensuring an adequate nutrient supply. Traditional processing techniques, such as drying, fermentation, salting, smoking, milling and cooking, have historically enhanced the preservation and digestibility of food while maintaining its recognisable form [1]. During the second half of the 20th century, profound changes in food production systems, global food markets and consumer lifestyles led to a progressive transformation of the food environment. Industrial food manufacturing shifted increasingly from preserving whole foods to formulating highly engineered products made from refined ingredients, industrially derived substances and multiple cosmetic additives designed to maximise palatability, convenience, shelf life and profitability. These products differ substantially from minimally processed foods in terms of nutrient composition, food matrix, physical structure, sensory characteristics, eating rate and biological effects and are classified as ultra-processed foods (UPFs) [2]. These changes have paralleled the global nutrition transition, characterised by a progressive shift from traditional dietary patterns towards diets rich in energy-dense, consumption of highly processed products, accompanied by sedentary lifestyles. While nutritional challenges in the early twentieth century were predominantly related to the risk of low food availability, to date populations are facing a double burden of malnutrition, characterized by both overnutrition, driven by the excessive availability and consumption of energy-dense foods, and undernutrition, resulting from poor dietary quality and the inadequate intake of essential nutrients despite sufficient or excessive caloric intake. These dietary changes are related to an increased risk of obesity and chronic non-communicable diseases, including type 2 diabetes, cardiovascular disease, metabolic dysfunction-associated fatty liver disease, cancer and neurobehavioural disorders. Although this transition has occurred worldwide, Mediterranean countries have experienced particularly rapid dietary changes, with a progressive erosion of traditional Mediterranean dietary habits and an increasing consumption of industrially UPFs, particularly among younger generations [3].

The evolution of food availability and consumption behaviors has progressively shifted toward environments that are metabolically mismatched with the current human genetic configuration, which has remained substantially unchanged for more than 20,000 years. At the same time, globalized food marketing tries to meet emerging sociocultural demands, such as ease of access and consumption, low price, and an increased demand for gratification, by continuously introducing UPFs characterized by high energy density and low nutritional value. This process generates a shift in responsibility from the individual to the institutional level, systematically steering food choices toward options that are detrimental to health. Indeed, there is growing epidemiological evidence that consistently associates higher UPFs consumption with an increased risk of obesity and several chronic diseases, considering socioeconomic inequalities, pediatric exposure and the progressive displacement of traditional dietary patterns [4–9]. In addition to recent reviews that have primarily focused on cardiometabolic outcomes associated with UPFs consumption [10–12], this Position Paper from the Italian Association of Dietetics and Clinical Nutrition (ADI) provides a comprehensive and critical appraisal of the available evidence on UPFs consumption and metabolic dysfunction, offering a broader comprehensive appraisal that extends to hepatic, oncological, and neurobehavioral outcomes. We also discuss biological mechanisms that could link UPFs to disease, with a particular focus on their possible role in contributing to mitochondrial dysfunction, in terms of compromised oxidative phosphorylation, reduced ATP production, altered redox homeostasis and excessive generation of reactive oxygen species [13]. The result is the consequent impairment of the metabolic flexibility, the ability to adapt substrate oxidation appropriately to nutrient availability and energy requirements by switching from carbohydrate to lipid utilization [14]. We also consider the potential link between UPFs consumption and immunometabolic regulation. The immunometabolism is defined as the interaction between metabolic pathways and immune cell function. In this process, metabolic reprogramming influences inflammatory responses and tissue homeostasis [15, 16]. Overall, although these mechanisms are considered biologically plausible, they are emerging pathways linking UPFs consumption to metabolic dysfunction rather than fully established causal processes in humans. Moreover, we critically address methodological challenges associated with the NOVA framework, thereby offering a more comprehensive and translational perspective. Finally, we summarise ADI’s official recommendations for clinical practice and public health.

## Methods

### Search Strategy

This narrative review was developed using a structured approach to the identification and selection the most relevant evidence on ultra-processed foods (UPFs) and metabolic health. A comprehensive search was conducted in PubMed/MEDLINE, Embase, Scopus, and Web of Science between January 2025 and January 2026. The literature search primarily covered studies published from January 2018 to January 2026, a timeframe selected to capture the rapid expansion of research following the widespread adoption of the NOVA classification and the substantial increase in prospective cohort studies, randomized controlled trials, systematic reviews, umbrella reviews, and mechanistic investigations published during recent years. The search strategy was tailored to each database using a combination of free-text terms and controlled vocabulary (MeSH/Emtree) with free-text keywords using Boolean operators. The core search syntax included combinations of: “ultra-processed foods or ultra processed foods,” “UPF,” “NOVA classification,” “food processing,” “metabolic health,” “metabolic flexibility,” “obesity,” “adiposity,” “insulin resistance,” “type 2 diabetes,” “cardiometabolic risk,” “inflammation,” “oxidative stress,” “mitochondrial function,” “microbiome,” “gut barrier,” “dysbiosis,” “metabolic-associated fatty liver disease/MAFLD,” “metabolic-associated steatohepatitis/MASH,” “cancer,” “depression,” “anxiety,” and “mental health.

The search strategies were adapted to suit the indexing system of each database. Reference lists from systematic reviews, umbrella reviews, meta-analyses, key cohort studies, randomised controlled trials and relevant position papers were manually reviewed to identify additional publications not detected by the electronic search. Publications from before 2018 were also included if they were seminal studies, introduced key concepts (e.g. the NOVA classification) or provided the first high-quality evidence of specific biological mechanisms or clinical associations.

### Inclusion and Exclusion Criteria

Studies were eligible for inclusion if they met one or more of the following criteria: (i) observational studies, including prospective cohort, case-control, and cross-sectional designs; (ii) randomized controlled trials and controlled feeding interventions; (iii) systematic reviews and meta-analyses; and (iv) consensus statements or methodological papers relevant to UPF definitions, classification systems such as NOVA, and exposure assessment. Both adult and pediatric populations were considered, provided that UPF exposure was explicitly defined was explicitly defined according to the NOVA classification or an equivalent processing-based framework. Studies addressing one or more of the following domains were considered: body weight or adiposity, obesity, insulin resistance or glycemic control or glucose metabolism, cardiometabolic disease, inflammatory biomarkers, inflammation and immunometabolism, mitochondrial function, gut microbiota and intestinal barrier integrity, liver-related outcomes, cancer, neurobehavioral or mental health outcomes.

Evidence was synthesized narratively, with particular emphasis placed on high-quality systematic reviews, umbrella reviews, randomized controlled trials, and large prospective cohort studies. Experimental and mechanistic studies were primarily used to provide biological plausibility and to interpret epidemiological findings rather than to infer causality. No formal risk-of-bias assessment was performed, in keeping with the narrative and position paper nature of the manuscript.

### Consensus Development and Approval Process

This document was developed as the official position statement of the Italian Association of Dietetics and Clinical Nutrition (ADI) via a structured expert consensus process. The group of authors prepared an initial draft based on a comprehensive assessment of the available scientific evidence. The final version was then peer-reviewed by ADI experts with proven expertise in clinical nutrition, dietetics and public health. The authors then critically discussed all comments and revision proposals until consensus was reached on the scientific content, the interpretation of the evidence, and the final wording of the position statements and recommendations. No formal Delphi procedure, quantitative voting process, or evidence-grading framework was applied. Instead, all experts reached unanimous agreement through iterative discussion. The final document was then reviewed and formally approved by the ADI Executive Board, thereby establishing the official position of the Italian Association of Dietetics and Clinical Nutrition.

## Consumption and Global Diffusion of Ultra-Processed Foods

The consumption of UPFs is now a global phenomenon, and it varies considerably across the different regions and the demographic groups. It is significantly influenced by socioeconomic, environmental and commercial factors. Urbanisation, limited time for mealtimes, low prices, aggressive marketing and digital food delivery platforms all contribute to increased exposure to UPFs. In Italy, although this consumption still accounts for a relatively modest proportion in terms of food weight (about 6% of total food purchases), it contributes substantially to the daily energy intake, providing up to 23% of the total calories consumed by adults and the elderly, with an upward trend observed as early as 2005. In the United States, UPFs represent more than 50% of daily caloric intake; in Canada approximately 48%; in Australia over 40%; and in Japan less than 30% [17, 18] Across European countries, the proportion of energy intake derived from UPFs averages around 27% of total caloric intake, with marked inter-country differences. In the United Kingdom and Sweden, UPFs may exceed 40–50% of total caloric intake (reaching over 60% among adolescents), whereas in Portugal, Romania, and Germany, the contribution remains below 40% [19].

Several evidence indicates that the proportion of daily calories derived from UPFs is particularly high in high-income countries and among younger age groups, contributing substantially to the progressive deterioration of dietary quality. The dietary habits of younger populations show greater preference on sweet and savoury snacks, sugar-sweetened beverages, and ready-to-eat meals. In addition, children and adolescents are particularly vulnerable because they are highly exposed to digital advertising, school food environments and products specifically designed to maximise palatability and brand loyalty. On the other hand, even if older generations tend to preserve dietary patterns more closely aligned with traditional food models, the increasing consumption of UPFs in mediterranean populations, is contributing to the progressive erosion of traditional dietary patterns [20]. This change is clinically significant because Mediterranean dietary patterns are characterised by minimally processed foods, high fibre intake, plant-based foods and lower dietary energy density. The early and repeated exposure to UPFs, particularly during childhood and adolescence, may exert long-term negative effects on metabolic health, amplifying the risk of obesity, insulin resistance, and other chronic non-communicable diseases [21, 22]. Marketing strategies targeting younger consumers, together with the inappropriate availability of UPFs within schools and educational environments, represent a profound inconsistency in public health practice [23].

## Food Classification and Definition of Ultra-Processed Foods

The NOVA food classification system currently represents the most widely used scientific framework for categorizing foods on the degree of industrial processing. The NOVA classification system it was developed to shift the focus from nutrient-based evaluation to processing-based assessment, NOVA emphasizes the structural, physical, and chemical transformations made to foods during the industrial food manufacturing process and how these foods are part of overall dietary patterns and their effects on health.

Based on this classification system, foods are classified into four groups:

- Group 1: Unprocessed or minimally processed foods
  This group includes all edible parts of plants or animals, as well as the foods that have undergone only to minimal alterations for the purpose of preservation or preparation (cleaning, removal of the inedible fractions, grinding, drying, pasteurization, refrigeration, freezing). These foods retain their original structure and nutritional profile. Fruits, vegetables, legumes, whole grains, fresh meat, fish, milk, and eggs belong to this group.
- Group 2: Processed food ingredients
  This group consists of substances extracted from the foods of the group 1 used to cook, prepare and season the foods (vegetable oils, butter, lard, sugar, honey, and salt).
- Group 3: Processed foods
  This group includes the foods of group 1 that have been processed by adding salt, sugar, and oil (ingredients from group 2) to improve their palatability and shelf life. Bread, cheeses, canned vegetables, and preserved foods fall into this category.
- Group 4: UPFs
  UPFs are industrial formulations composed of multiple ingredients not commonly used in home cooking. These foods undergo chemical modifications through a series of industrial processes such as hydrogenation and hydrolysis and contain additives such as flavourings, colorants, emulsifiers, stabilizers, thickeners, artificial sweeteners, and other functional molecules that mimic sensory properties and produce a hyperpalatability effect. Indeed, the primary goal of these formulations is to improve sensory appeal, shelf stability, and commercial profitability [22, 23].
  This category of UPFs includes packaged snacks, sugary drinks, industrial baked goods, breakfast cereals, ready-to-eat meals, fast food, processed meat products, confectionery, and many foods specifically intended for infants and children [22, 23].

The NOVA classification has significant limitations. It may vary depending on the culture, country and dietary assessment tools used. Inter-observer variability may also occur when ingredient lists are incomplete or ambiguous. Furthermore, NOVA does not provide quantitative thresholds for nutrients, additives, or the degree of processing [24]. It should also be recognised that not all foods in NOVA Group 4 are metabolically equivalent. Ultra-processed foods (UPFs) constitute a heterogeneous category, encompassing products that differ substantially in energy density, fibre content, protein quality, added sugars, fat composition and in alteration degree of the food matrix [25]. Therefore, the health impact of UPFs should be interpreted in relation to processing and overall nutritional quality, as discussed in more detail below.

## The Biological Basis for Considering Ultra-Processed Foods as a Distinct Food Category

A key issue in the NOVA classification is whether the health risks associated with ultra-processed foods (UPFs) are due to their low nutritional quality, or if the industrial processing itself has additional biological effects. The UPFs contain high levels of added sugars, sodium and saturated fats and low levels of dietary fibre, but these products cannot be considered metabolically equivalent to minimally processed foods with a similar macronutrient profile. Unlike traditional food processing, ultra-processing breaks down raw materials extensively into purified constituents (e.g. starches, protein isolates, refined oils and sugars), which are then recombined with cosmetic additives to create formulations designed specifically to ensure convenience, shelf stability, texture and sensory appeal [26]. These industrial processes alter the original food matrix profoundly, changing the physical organisation of nutrients and affecting their digestion, absorption, and metabolism (see below). The breakdown of the food matrix can accelerate nutrient bioavailability and gastric emptying, leading to higher postprandial glycaemic, insulinemic, and lipaemic peaks than are observed after consuming structurally intact foods [26]. Furthermore, UPFs generally require less chewing, provide reduced oral sensory exposure, and are consumed at significantly faster rates. Experimental evidence indicates that these characteristics reduce physiological satiety signals, decrease oral processing time, and encourage passive overeating [27].

Another distinctive feature of UPFs is their engineered hyperpalatability, which is achieved by combining refined carbohydrates, fats, salt, flavourings, emulsifiers, and texturising agents in specific ways to enhance food reward and encourage repeated consumption beyond homeostatic energy requirements. Taken together, these characteristics suggest that the metabolic effects of UPFs may stem not only from their nutritional composition, but also from the interaction between food structure, industrial formulation, sensory properties and eating behaviour.

Therefore, the NOVA classification should be considered a complementary framework that incorporates dimensions of food quality, such as matrix integrity, industrial fractionation and formulation, that are not captured by conventional nutritional profiling systems, rather than an alternative to nutrient-based dietary assessment.

NOVA classification presents some practical challenges for the consumers. It should be noted that, it is not always easy to distinguish between processed and ultra-processed products, particularly in the absence of standardized and clear labels. Consumers should carefully read and analyse the full list of ingredients. In this group we found products containing more than five ingredients, especially when including additives such as lactose, casein, gluten isolates, whey derivatives, hydrogenated or interesterified fats, flavour stabilizers, hydrolyzed proteins, corn syrup, high-fructose corn syrup, invert sugar, maltodextrins, dextrose, modified starches, or ingredients labelled with “E” numbers. In addition, the position of such ingredients in the list is relevant, as components are listed in descending order by weight, and the presence of cosmetic additives among the first ingredients suggests a substantial formulation.

However, certain micronutrients added for fortification, such as calcium, iron, thiamine, niacin, ascorbic acid, folic acid, or vitamin D, should not be interpreted as indicators of ultra-processing, as fortification may occur across different food categories. Similarly, processing aids such as corn starch may be used in both minimally processed and ultra-processed products.

Quantitative measures of the nutritional quality, caloric density, or specific nutrient thresholds are not directly take into account in the NOVA system. As a consequence, foods with similar nutritional profiles may be classified differently based solely on their level of processing, and some industrial products with high nutritional value may be classified as ultra-processed despite their favorable composition in terms of macro- and micronutrients. Conversely, some minimally processed foods may have a high energy density without being classified as UPFs [28, 29].

UPFs are rich in interesterified fats and invert sugar. The first ones are produced through a chemical or enzymatic rearrangement of the fatty acids within the triglyceride molecules, avoiding the formation of trans double bonds since extensive scientific evidence has demonstrated their negative metabolic effects [30]. For this reason, they are subject to strict legal restrictions or even banned in many countries and the World Health Organization has recommended eliminating these trans fats from the global food supply. Instead, the interesterified fats do not contain trans isomers, however the long-term effects of their consumption on metabolic health have not yet been extensively studied in humans and the results available to date are contradictory [28, 29]. Invert sugar, obtained by the hydrolysis of the sucrose into glucose and fructose, enhances sweetness, solubility, and moisture retention, and improve the texture, the stability, and the shelf life of UPFs. Notably, metabolically, the presence of free monosaccharides induces the rapid glucose absorption and the increase of glycaemia. High levels of fructose, particularly in the context of high-calorie diet, have been linked to the activation of de novo lipogenesis in the liver, the development of insulin resistance, and the accumulation of ectopic fat [31, 32].

Taken together, these findings demonstrate how technological modifications aimed at optimizing functional and sensory properties of foods can influence the regulation of metabolic pathways relevant to long-term health outcomes.

## Strengths and Weaknesses of the NOVA System

The classification of foods described above, which is based mainly on the level of processing could lead to confusion between the degree of food processing and their nutritional quality. Several concerns have been raised regarding potential classification errors, a limited reproducibility due to varying assessment tools, and the absence of quantitative thresholds that allow us to distinguish the processed from ultra-processed foods. Products that have been reformulated to achieve a better nutritional profile could still be classified as ultra-processed, while minimally processed foods with high energy density may not be detected by the system. NOVA system does not directly incorporate nutritional density indices and standardization of the portions, potentially limiting its accuracy when used as the sole indicator of food quality. However, the NOVA classification is able to highlight the structural characteristics of industrial food formulations (alterations to the food matrix, the use of cosmetic additives, and the engineering of hyper-palatability), aspects that are not adequately considered by models based solely on nutrient analysis. The NOVA system should be considered a complement to, rather than an alternative to, nutrient-based approaches. A combined assessment of food processing, nutritional profile, dietary habits and food matrix can provide a more comprehensive evaluation of food quality.

## Additives, Potentially Toxic Substances, and Cumulative Effects

UPFs are characterized by a long and complex list of ingredients containing numerous additives authorized by law and evaluated according to toxicological frameworks, including acceptable daily intake and dose–response assessment. However, current risk assessment is generally based on individual compounds, whereas habitual UPFs consumption may expose consumers to multiple additives and processing-derived compounds simultaneously. Although individual substances generally comply with established safety limits, the critical issue lies in cumulative exposure. Habitual and repeated consumption of multiple UPFs throughout the day results in exposure that can significantly exceed the safety thresholds for the intake of individual compounds and can trigger effects with an impact greater than the simple algebraic sum of the individual toxicities. However, the potential cumulative or combined effects of these exposures remain insufficiently characterised and this phenomenon, known as the cocktail effect, is not sufficiently addressed in current food risk assessment frameworks [33, 34]. Although this effect should be considered a hypothesis of concern rather than a fully established mechanism in humans, several studies have revealed potential correlations between these dietary additives and their impact on health. Among the substances that can alter the integrity of the intestinal barrier, induce dysbiosis and compromising glucose homeostasis we found the emulsifiers (e.g., carboxymethylcellulose E466 and polysorbate 80 E433) [35, 36] and intense sweeteners (e.g., sucralose E955, acesulfame K E950), that have been linked to gut microbiota and metabolic changes mainly in experimental and selected human studies [37–39]. The formation of potentially carcinogenic compounds is attributable to the presence of nitrites and nitrates (sodium nitrite E250). Flavour enhancers (monosodium glutamate E621) may contribute to overeating since they are designed to increase palatability. Metabolic alterations may also be caused by the presence of colorants (ammonium caramel E150c/E150d) that contain processing contaminants such as 4-methylimidazole (4-MEI), polycyclic aromatic hydrocarbons, heterocyclic amines, advanced glycation end products (AGEs), added phosphates (found in beverages and processed meats) and other contaminants generated during high-temperature treatments and refining (including acrylamide found in baked products and snacks), [40, 41]. In particular, AGEs and thermal contaminants are biologically plausible contributors to oxidative stress [42, 43] and packaging-related products are a source of numerous chemical substances, several of which have documented endocrine disruptors effects on human health [44–46]. Table 1 provides an overview of these categories of substances, specifying their technological function and their potential metabolic implications.

**Table 1:** Selected technological ingredients and processing-derived compounds commonly found in ultra-processed foods and their potential metabolic implications

Category	Examples	Technological Function	Food Sources	Potential Metabolic Implications	Evidence Source
Food Additives	Emulsifiers (E466 carboxymethylcellulose, E433 polysorbate 80), flavor enhancers (E621 monosodium glutamate), artificial sweeteners (sucralose E955, acesulfame K E950)	Improve texture, stability, palatability, and shelf life	Processed sauces, packaged snacks, fast food, soft drinks	Dysbiosis, increased intestinal permeability, low-grade inflammation, altered glucose homeostasis	Limited human intervention evidence [47]; supported by animal in vivo [48] and human microbiota ex vivo studies [36].
Preservatives	Sodium nitrite (E250), sodium nitrate (E251), sulfites, potassium sorbate	Prevent microbial growth and extend shelf life	Processed meats, cured products, packaged bakery items	Formation of reactive compounds (e.g., nitrosamines), oxidative stress, potential carcinogenic pathways	Human intervention evidence based primarily on biomarkers of endogenous N-nitroso compound formation [49]; supported by animal in vivo [50] and in vitro mechanistic studies [51]. Evidence for long-term effects of individual preservatives remains limited.
Processing-Derived Compounds	Advanced glycation end products (AGEs), acrylamide, polycyclic aromatic hydrocarbons (PAHs)	Generated during high-temperature processing (frying, roasting, extrusion)	Fried foods, baked products, snacks, processed cereals	Oxidative stress, inflammation, mitochondrial dysfunction, endothelial damage	Human intervention evidence for dietary AGEs [52], supported by animal in vivo [53] and in vitro mechanistic studies [54].
Interesterified Fats	Chemically or enzymatically rearranged triglycerides (often using palm, soybean, or stearic acid–rich fats)	Modify melting properties and texture; alternative to trans fats in processed foods	Margarines, baked products, confectionery, spreads	Possible altered lipid and glucose metabolism; long-term metabolic effects still under investigation	Limited human intervention evidence [55], supported by animal in vivo studies [56]. Human findings are heterogeneous, and long-term clinical evidence remains insufficient.
Invert Sugars / Rapidly Absorbable Sugars	Invert sugar (glucose + fructose), high-fructose corn syrup, glucose syrups, maltodextrins	Enhance sweetness, hygroscopicity, and product stability	Soft drinks, confectionery, breakfast cereals, baked products	Rapid glycemic spike, increased hepatic de novo lipogenesis, insulin resistance, ectopic fat accumulation	Human intervention [57] and prospective observational evidence [58], supported by animal in vivo mechanistic studies [32].

## Food Addiction and Artificial Enhancement of Taste

UPFs are specifically designed to maximise sensory gratification through carefully crafted combinations of sugars, fats, salt, flavourings and texturising agents. This combination is calibrated to reach the so-called *bliss point* that is the level of palatability able to maximise consumer’s appeal. This artificial enhancement of taste represents the main feature of industrial formulations, aimed at increasing over consumption and market competitiveness. These products often combine high-glycaemic-index carbohydrates with rapidly absorbable fats and sodium in matrices that facilitate rapid oral-sensory stimulation and post-ingestive gratification [59]. The hyperpalatability of the UPFs interferes not only with the physiological mechanisms that regulate appetite and satiety, but also with the reward processing [60]. Indeed, the consumption of UPFs overstimulates mesolimbic dopaminergic reward pathway, particularly within the ventral tegmental area and nucleus accumbens circuits, promoting hedonistic eating behaviors dissociated from homeostatic energy needs. The prolonged activation of these neural circuits leads to reduced availability of the dopamine receptors, resulting in a reduced sensitivity to reward and to a compensatory increase in food intake to achieve an equivalent hedonic response. At the same time, the disruption of gut-brain signaling and the altered secretion of hormones such as GLP-1, PYY, leptin, and insulin can impair normal appetite regulation, further promoting over-eating [45, 46]. Neuroimaging studies have demonstrated an altered activation pattern in brain regions associated with reward and executive control in individuals consuming a big quantity of UPFs or highly palatable foods [45, 46]. These effects are significant in children and adolescents due to the ongoing maturation of the prefrontal cortical regions involved in executive functions [61]. Early and continuous exposure to UPFs can influence taste preferences towards highly sweet and salty foods. This phenomenon can compromise taste education, promoting a long-term preference for high-energy-density industrial products and reducing the consumption of fruit and vegetables and whole foods [62]. Furthermore, the rapid digestibility of many UPFs can lead to rapid glucose absorption and consequently high postprandial blood glucose fluctuations, which in turn interact with reward signals and dysregulate homeostatic eating patterns [20]. Understanding these neurobiological and behavioural mechanisms is essential the evaluate the broader impact of UPFs on eating habits, the risk of obesity and long-term metabolic health [45, 46, 62]. The concept of food addiction remains scientifically debated. While some highly palatable UPFs may activate reward-related neural circuits and have been linked to compulsive-like eating behaviours in susceptible individuals, the extent to which this phenomenon should be considered analogous to substance addiction remains controversial. Therefore, in this paper the term “food addiction” is used cautiously to describe a behavioural phenotype characterised by loss of control, craving and persistent intake despite negative consequences, rather than as a universally accepted diagnostic category.

## Ultra-Processed Foods and Health Implications

Higher UPFs consumption appears to be associated with increased all-cause mortality, particularly among high consumers of ready to eat meat or poultry-based dishes and sugar sweetened or artificially sweetened beverages. The excessive intake of ultra-processed dairy based desserts and breakfast foods have also been associated with increased mortality risk. Specifically, mortality rates are estimated to be 4% higher among high consumers (mean intake of 7.4 servings/day) compared with low consumers (approximately 3 servings/day) [63].

A 10% increase in UPFs consumption corresponds to a 3% increase in mortality risk among individuals aged 30–69 years. In the United States, where average UPFs intake exceeds 50% of total energy, approximately 14% of premature deaths are estimated to be attributable to an excessive UPFs consumption [64, 65].

High UPFs intake have been associated with increased risk of cardiovascular disease (+ 17%), coronary heart disease (+ 23%), stroke (+ 9%), cancer, metabolic disorders (type 2 diabetes), anxiety, and dementia. Some UPFs are easily identifiable (e.g., candies, soft drinks, processed meat, poultry, and fish products such as bacon, hot dogs, breaded fish sticks, chicken sausages), whereas others are less readily recognized (e.g., some industrial whole-grain breads) [66].

Diets minimizing the UPFs consumption have been associated with greater weight loss compared with diets in which approximately two-thirds of energy intake is derived from UPFs [67]. Additional systematic reviews and meta-analyses have linked UPFs consumption to chronic degenerative diseases, including type 2 diabetes, non-alcoholic steatohepatitis, inflammatory bowel disease, and sarcopenia [68–70]. Packaging materials also warrant consideration, as plastic containers and multilayer packaging may release harmful chemical contaminants such as phthalates and bisphenols that affect health [44]. It is important to note that some of the available evidence on the relationship between UPFs consumption and chronic disease risk comes from observational studies. Therefore, residual confounding cannot be ruled out. UPFs intake is often associated with lower socioeconomic status, lower educational level, smoking, reduced physical activity, shorter sleep duration, higher psychological stress, and poorer overall dietary quality. These factors may contribute to the observed associations to some extent and should be taken into account when interpreting the strength of causal inference [71]. The health effects associated with UPFs are summarized below according to a common framework that include epidemiological evidence and proposed biological mechanisms.

### UPFs and Chronic Inflammation

**Fig. 1:**
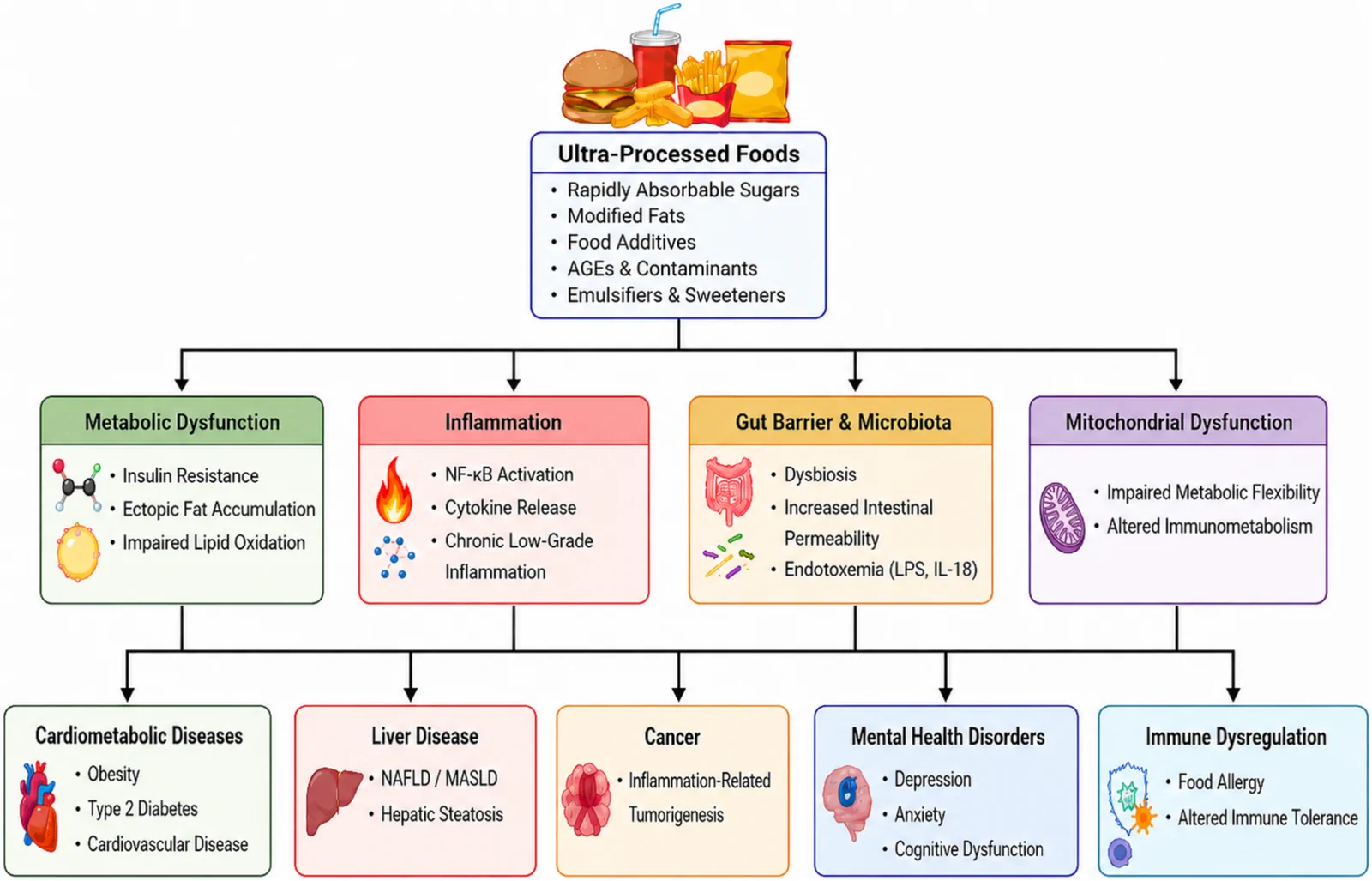
UPFs consumption and non-communicable chronic diseases: role of mitochondrial dysfunction. The high consumption of ultra-processed foods (UPFs) is associated with the risk of non-communicable diseases since expose the individuals to high levels of free glucose and fructose, trans and intersterified fats, food additives, processing-derived compounds such as emulsifiers and sweeteners, and advanced glycation end products (AGEs). All these alter the mitochondrial function leading to metabolic inflexibility and oxidative stress. The impairment of these mechanisms has been linked to metabolic dysfunction, chronic low-grade inflammation, gut barrier disruption, neuroendocrine and immune dysregulation resulting in an increased risk of cardiometabolic and liver disease, cancer, mental health disorders, and immune system disorders. Figure created with BioRender.com

The excessive consumption of UPFs is associated with a dysregulation of the hypothalamic–pituitary–adrenal axis. Frequent consumption of high-glycemic-load foods induces a rapid increase of the levels of blood glucose and consequent hyperinsulinemia. This process is followed by a rapid drops in glycemia that trigger counter-regulatory stress responses mediated by cortisol, adrenaline, and noradrenaline that promote visceral adiposity, lean body mass loss, and impaired insulin sensitivity [72–74]. UPF-rich dietary patterns have been associated with chronic low-grade inflammation and may contribute to mitochondrial alterations through multiple mechanisms. [75]. Mitochondrial dysfunction is thought to be one of the main pathways through which poor diet quality leads to metabolic diseases. However, direct evidence from human studies on the consumption of ultra-processed foods (UPFs) remains limited. Notably, in animal models, UPF-rich diets lead to a dysregulation of pathways mediated by AMPK and mTOR, key cellular energy sensors, with a consequent reduction of mitochondrial fatty acid β-oxidation [76]. Impaired lipid oxidation compromises metabolic flexibility, the ability to efficiently switch between carbohydrate and lipid oxidation, favouring persistent hyperinsulinemia and glycemic instability [33, 77] (Fig. 1).

The chronic consumption of UPFs also alters the gut microbiota, resulting in reduced microbial diversity, and increase the intestinal permeability exacerbating systemic inflammation and worsens mitochondrial and metabolic dysfunctions [78] (Fig. 1). Over time, chronic inflammation, mitochondrial dysfunction, impaired β-oxidation, and gut dysbiosis interact to promote the accumulation of fat mass, particularly visceral fat, as well as the loss of lean mass, and deterioration of muscle quality. These features are consistently associated with the development of metabolic dysfunctions that are characteristic of adulthood, including insulin resistance, metabolic sarcopenia, and increased cardiometabolic risk [79, 80] (Fig. 1). However, direct human evidence remains limited, and further longitudinal, mechanistic, and intervention studies are needed to establish causality and disentangle the relative contribution of food processing, food additives, and nutrient composition to the observed cardiometabolic effects. As recently highlighted, the current body of evidence strongly supports an association between high UPF consumption and cardiometabolic risk, while further mechanistic research is required to strengthen the evidence base and inform targeted nutrition policies [81].

### UPFs and Cancer Risk

Several studies have demonstrated that the excessive consumption of UPFs is related to an increased risk of developing several types of cancer (colorectal adenomas and carcinomas, breast cancer, and pancreatic cancer) [82–84]. In detail, the high intake of potassium sorbate has been associated with a 14% increase in overall cancer risk and a 26% increase in breast cancer risk. The intake of sulfite has been associated with a 12% increase in overall cancer risk. Sodium nitrite assumption has been linked to a 32% increased risk of prostate cancer, while potassium nitrate intake has been associated with increased risk of overall cancer (13%) and breast cancer (22%). Total acetates have been associated with a 13% increase in overall cancer risk and a 25% increase in breast cancer risk, while acetic acid intake has been associated with a 12% increase in overall cancer risk. Among antioxidant preservatives, only total erythorbates, and sodium erythorbate in particular, have been associated with increased cancer incidence [85].

The exposure to food additives and contaminants resulting from food processing (such as acrylamide and polycyclic aromatic hydrocarbons), and the accumulation of AGEs could activate pro-inflammatory and pro-oncogenic pathways, with a consequent onset of a state of low-grade chronic inflammation and increased oxidative stress. In addition, the alterations in the gut microbiota, which are frequently observed following the consumption of diets rich in UPFs, could further compromise the integrity of the intestinal barrier, exacerbating systemic inflammation and triggering immune responses [86] (Fig. 1).

Although epidemiological studies consistently report positive associations between high UPFs consumption and the risk of several cancers, most evidence derives from observational cohorts and residual confounding cannot be excluded. Furthermore, many of the proposed biological mechanisms are supported primarily by experimental and mechanistic studies rather than direct human evidence. Therefore, while limiting UPFs consumption represents a reasonable public health strategy, further prospective and intervention studies are needed to clarify causal relationships and identify the contribution of specific food processing characteristics, additives and contaminants.

### UPFs and Neuroinflammation

Epidemiological evidence points to an association between diets rich in UPFs and more unfavourable psychological outcomes, in particular depression, anxiety, and mood disorders, especially during critical periods of neurobiological development such as adolescence and early adulthood [87].

The proposed mechanisms are systemic and multifactorial. The altered regulation of the gut-brain axis can induce chronic low-grade inflammation due to an increased intestinal permeability that facilitate the translocation of pro-inflammatory metabolites and reduce the production of short-chain fatty acids, beneficial neuroactive compounds [78, 87]. Furthermore, the altered glucose metabolism associated with UPFs consumption may further interfere with the synthesis and release of serotonin and dopamine, key neurotransmitters for the mood regulation, and may contribute to emotional instability. Ultimately, the persistently elevated cortisol levels, may impair the emotional regulation mechanisms [88]. These mechanisms that include gut–brain axis alterations, low-grade inflammation and oxidative stress suggest that UPFs consumption may contribute to adverse neurobehavioural outcomes. However, current evidence is largely based on observational epidemiological studies and experimental models, making it difficult to establish causality or exclude residual confounding and reverse causation. Additional longitudinal and intervention studies are therefore warranted to clarify these relationships. [89] (Fig. 1).

### UPFs and Food Allergy: Mitochondrial and Immunometabolic Mechanisms

Several studies suggest that the high consumption of UPFs may contribute to immune dysregulation and allergic diseases through the alterations of immunometabolic mechanisms [90]. Mitochondria play a central role in immunometabolism, regulating immune cell activation, differentiation, and function. Alterations in mitochondrial capacity and disruptions in redox balance can influence the metabolic programming of immune cells, modulating inflammatory and allergic responses. In particular, the increased mitochondrial ROS production and impaired oxidative phosphorylation may promote a shift toward pro-allergic Th2 immune responses and disrupt the mechanisms of immune tolerance [91]. Indeed, in a murine model of food allergy it has been demonstrated an association between the hepatic mitochondrial dysfunction and oxidative stress, with elevated expression of Th2 cytokines, suggesting a link between immune activation and mitochondrial metabolic alterations [91, 92].

It has been also demonstrated that dietary intake of AGEs, which are abundant in many UPFs, may contribute to allergic sensitization by disrupting mitochondrial function, redox balance and immune homeostasis. In vitro and clinical investigations have shown that the exposure to AGEs impairs intestinal barrier integrity, increases transepithelial passage of food antigens, induces oxidative stress and inflammation, and promotes a Th2-mediated immune response. Furthermore, a significantly higher dietary AGEs intake, with increased skin accumulation of AGEs were found in children with food allergy compared with healthy controls [13, 16].

Overall, these findings support the biological plausibility that diets characterized by high UPFs consumption may influence immune tolerance through mitochondrial dysfunction, oxidative stress and gut barrier alterations. Nevertheless, current evidence remains limited, with mechanistic data deriving predominantly from animal and in vitro studies, whereas epidemiological evidence in humans is still scarce. Further prospective human studies are required before causal relationships can be established.

## Clinical Nutrition Practice

Assessment of UPFs intake may be integrated into routine clinical nutrition practice as part of a comprehensive dietary evaluation. This approach is particularly relevant in obesity management, diabetes prevention, cardiometabolic risk assessment, paediatric nutrition, MASLD, frailty, sarcopenia and hospital-based clinical nutrition. Clinicians should evaluate UPFs intake taking into account the total food intake (quantity, g- energy density-kJ), dietary pattern, fibre intake, protein quality, added sugars, fat quality, meal timing and socioeconomic context. A practical approach may include asking patients about the frequency of packaged snacks, sugar-sweetened beverages, industrial baked products, ready-to-eat meals, processed meats, breakfast cereals and fast foods. Nutritional counselling should focus on gradual replacement rather than simple prohibition. Patients should be encouraged to replace UPFs with minimally processed alternatives, including fruit, vegetables, legumes, whole grains, nuts, fish, eggs, milk and yogurt without added sugars, and home-prepared meals based on Mediterranean dietary principles.

**Fig. 2:**
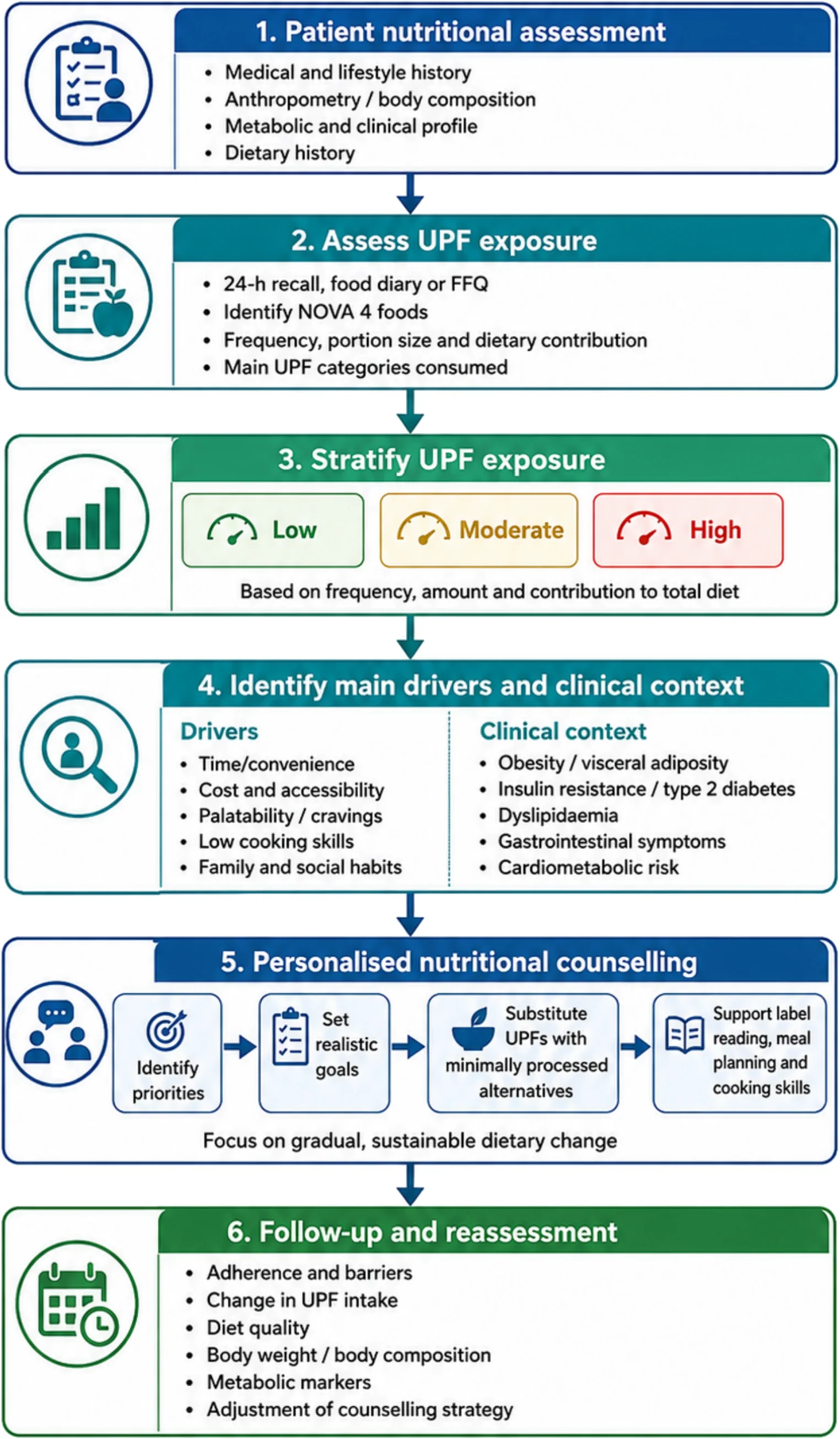
Clinical algorithm for the assessment of ultra-processed food exposure and its integration into nutritional counselling

In vulnerable patients, including older adults, individuals with cancer, sarcopenia, frailty, inflammatory bowel disease or metabolic syndrome, the reduction of UPFs should be balanced with the need to maintain adequate energy and protein intake. Therefore, individualised nutritional planning remains essential. In healthcare institutions, hospitals and long-term care facilities, UPFs reduction should be incorporated into procurement policies, menu planning and nutritional quality standards, while ensuring food safety, palatability, accessibility and adequate nutritional density. Practical clinical algorithm for UPFs assessment is reported in Fig. 2.

## Socioeconomic and Health Aspects: Prevention Measures and Actions to Reduce UPFs Consumption

The relatively low cost, widespread availability and aggressive marketing of UPFs have contributed to their increasing consumption worldwide, particularly among socioeconomically disadvantaged populations. While these products may improve food accessibility and shelf life, consuming large quantities of them raises important public health concerns as they often replace nutrient-dense, minimally processed foods in people’s diets. Furthermore, evidence regarding the long-term health effects of chronic UPFs consumption is largely derived from observational studies. Additional longitudinal and intervention studies are needed to strengthen causal inference. From a nutritional perspective, while UPFs can satisfy energy requirements, they often provide inadequate levels of the essential nutrients and bioactive compounds required to maintain optimal physiological function [93]. Consequently, dietary patterns characterised by high UPF consumption may lead to nutritional imbalances and increase the risk of chronic non-communicable diseases, particularly among vulnerable populations, thereby exacerbating existing health inequalities [94, 95].

## Limitations

Residual confounding remains an important limitation, as observational studies cannot fully account for all potential confounders, including socioeconomic status, lifestyle behaviours, and overall dietary patterns that may independently influence health outcomes. Additional methodological challenges arise from the considerable heterogeneity in the definition and classification of UPFs across studies. Although the NOVA classification system is widely adopted, its application is not always consistent, and differences in food categorisation may reduce comparability between studies and introduce classification bias. Dietary assessment methods also represent a potential source of bias. Most studies relied on self-reported dietary data, which are inherently prone to recall bias and measurement error. Another important limitation is the lack of long-term randomised controlled trials evaluating the health effects of UPFs.

Disentangling the effects of food processing from those of nutritional composition remains a major methodological challenge. Despite these limitations, the consistency of findings across diverse populations, study designs, and health outcomes strengthens the overall evidence linking higher UPF consumption with adverse health outcomes. Future research, particularly well-designed long-term randomised controlled trials and mechanistic studies, is needed to clarify the independent contributions of food processing and nutritional composition to disease risk and overall health.

Although studies demonstrating a direct association between UPF consumption and human health outcomes are still limited, the available evidence is rapidly growing. Therefore, this position paper also aims to encourage further scientific research and well-designed human studies to confirm and strengthen the evidence discussed, while raising public awareness and encouraging people to limit their consumption of these products.

## Recommendations and Key Messages

The Italian Association of Dietetics and Clinical Nutrition (ADI) reaffirms that reducing exposure to UPFs should be considered a relevant clinical and public health objective, especially in children, adolescents, and other vulnerable population groups. Evidence indicates that UPFs should not be regarded as interchangeable with their home-prepared or minimally processed counterparts, because they differ not only in nutrient profile, but also in food matrix, sensory engineering, eating rate, additive content, and metabolic effects [95–97].

Accordingly, ADI supports the following recommendations:

- A substantial reduction in habitual UPF consumption, within a whole-diet approach based on fresh and minimally processed foods.
- The integration of UPF-oriented guidance into nutritional counselling, obesity prevention strategies, and dietary recommendations, alongside conventional nutrient-based advice.
- The promotion of food and label literacy, enabling consumers to identify industrial formulations characterized by long ingredient lists, cosmetic additives, and ingredients of rare culinary use.
- The protection of children and adolescents through stricter regulation of marketing, digital advertising, and commercial promotion of UPFs directed at minors.
- The progressive reduction or exclusion of UPFs in schools, hospitals, universities, and other public catering settings, while increasing the availability of minimally processed alternatives.
- The adoption of front-of-pack interpretive labelling, clear procurement standards, and other food-environment measures that facilitate healthier default choices.
- The consideration of fiscal and regulatory policies, including taxes, marketing restrictions, and incentives that improve access to fresh, minimally processed foods, particularly for lower-income households.

These recommendations should not rely on individual responsibility alone. A coherent response requires coordinated action involving healthcare professionals, public institutions, educators, researchers, retailers, food service settings, and policy makers, because UPF consumption is shaped by price, availability, convenience, marketing pressure, and the broader food environment [95, 97]. In light of these considerations, we propose an algorithm presented as a flowchart to help professionals assess UPF intake using a 24-h recall, food diary or food frequency questionnaire (FFQ), and develop a nutrition counseling plan aimed at reducing their consumption (Fig. 2).

 **Box 1. ADI Position Statements on Ultra-Processed Foods**

1. Reducing the consumption of ultra-processed foods (UPFs) is a goal that should be considered clinically and in terms of public health, particularly for children, adolescents and other vulnerable groups.
2. UPFs are not nutritionally or biologically interchangeable with home-cooked or minimally processed foods. The differences extend beyond nutritional composition to include texture, processing level, sensory characteristics, eating behaviour, additives and metabolic effects.
3. Dietary counselling should include guidance on limiting UPFs consumption alongside traditional nutrient-based recommendations within the context of healthy eating patterns based primarily on fresh and minimally processed foods.
4. Understanding of food labels should be a public health priority to enable consumers to recognise ultra-processed foods and make informed choices about what they eat.
5. Children and adolescents should be protected from commercial exposure to UPFs, including marketing, digital advertising and other promotional strategies targeting minors.
6. Public institutions should progressively improve food environments by increasing access to minimally processed foods in schools, hospitals, universities and other public food service facilities.
7. Population-level policies should encourage healthier food choices through science-based measures, such as fiscal and regulatory policies that improve the accessibility and affordability of minimally processed foods.
8. Reducing the consumption of UPFs requires coordinated and multisectoral action. This responsibility should not fall solely on individuals, but rather be shared among healthcare professionals, educators, researchers, policymakers, retailers and food service providers.

## Conclusions and Institutional Position

UPFs represent more than a simple issue of poor food quality: they embody a broader alteration of the food environment that may adversely affect metabolic regulation, feeding behaviour, inflammatory pathways, and long-term disease risk. The available evidence, while heterogeneous across outcomes, suggests that dietary patterns high in UPFs are associated with less favorable weight-related and metabolic health trajectories than dietary patterns based on minimally processed foods [95]. However, it should be acknowledged that much of this evidence remains observational, and causal relationships have not been definitively established. Emerging evidence from mechanistic and short-term intervention studies provides support for potential biological pathways, though long-term randomised controlled trials are still lacking. From an institutional perspective, ADI considers the reduction of UPF consumption a reasonable public health priority and a potential target for prevention policies, while recognizing existing evidence gaps. In this framework, the responsibility for change should not be placed exclusively on individuals, since food choices are strongly conditioned by commercial determinants, retail environments, affordability, and pervasive marketing practices. ADI therefore supports a multi-level strategy that combines nutritional education, protection of children, healthier school and healthcare food environments, transparent labelling, responsible marketing regulation, and structural policies capable of reorienting food systems toward health-promoting options. Particular emphasis should be placed on promoting minimally processed dietary patterns and Mediterranean dietary traditions, which represent well-established models of healthy eating with extensive supporting evidence.

This position is consistent with the need to frame UPFs as a distinct and actionable construct within dietary guidance and obesity prevention, complementing rather than replacing traditional nutrient-based recommendations. However, further research is needed to better delineate the independent contributions of food processing versus nutritional composition, and to clarify which aspects of ultra-processing may be most relevant to health outcomes.

## Key References

- Monteiro CA, Louzada ML, Steele-Martinez E, et al. Ultra-processed foods and human health: The main thesis and the evidence. The Lancet. 2025;406:2667–2684.
  - This important review summarises the conceptual framework underlying ultra-processed foods (UPFs) and critically analyses the epidemiological and mechanistic evidence linking exposure to UPFs with adverse health outcomes. It is a key reference for understanding the broader public health implications of food processing beyond nutritional composition.
- Lane MM, Gamage E, Du S, et al. Ultra-processed food exposure and adverse health outcomes: Umbrella review of epidemiological meta-analyses. BMJ. 2024;e077310.
  - This umbrella review synthesises data from several meta-analyses, representing one of the highest levels of epidemiological synthesis currently available. The study confirms the consistency of associations between ultra-processed food consumption and numerous chronic diseases and mortality outcomes.
- Dicken SJ, Jassil FC, Brown A, et al. Ultraprocessed or minimally processed diets following healthy dietary guidelines on weight and cardiometabolic health: A randomized, crossover trial. Nat Med. 2025;31:3297–3308.
  - This randomised crossover study is important because it evaluates the direct metabolic effects of food processing under controlled conditions, rather than simply observing associations. The results will inform the ongoing debate about whether food processing itself has health effects that are independent of nutritional content.
- Maki KA, Sack MN, Hall KD. Ultra-processed foods: Increasing the risk of inflammation and immune dysregulation? Nat Rev Immunol. 2024;24:453–454.
  - This in-depth article explores the growing importance of UPFs in promoting inflammatory signalling and immune dysregulation. Its significance lies in its ability to link metabolic dysfunction to immune and inflammatory pathways that may underlie various non-communicable diseases.

## Data Availability

No datasets were generated or analysed during the current study.
