# Correction: Ultra-Processed Foods and Metabolic Dysfunction: Mechanisms, Clinical Implications, and Public Health Perspectives. Position Paper of the Italian Association of Dietetics and Clinical Nutrition (ADI)

**DOI:** 10.1007/s13668-026-00803-y

**Published:** 2026-10-05

**Authors:** Giovanna Trinchese, Barbara Paolini, Fabiano Cimmino, Filippo Valoriani, Lucio Lucchin, Carmela Bagnato, Annarita Sabbatini, Massimiliano Petrelli, Pietro Losignore, Francesca Marino, Giulia Tavella, Odette Misa Sonia Hassan, Annalisa Maghetti, Maria Grazia Carbonelli, Sebastiano Banni, Luigi Barrea, Maria Pina Mollica

**Affiliations:** 1 Department of Biology, University of Naples Federico II, Naples, Italy; 2 Italian Association of Dietetics and Clinical Nutrition (ADI), Rome, Italy; 3 Azienda Ospedaliera Universitaria Senese, Siena, IT Italy; 4 Azienda Ospedaliero Universitaria di Modena-Policlinico, Modena, Italy; 5 Dietetics and Clinical Nutrition, Bolzano Health District, Bolzano, Italy; 6 UOSD Clinical Nutrition and Dietetic, Hospital Matera, Matera, Italy; 7 Dietetic and Clinical Nutrition Unit, IEO European Institute of Oncology IRCSS, Milan, Italy; 8 Endocrinology and Metabolic Disease Clinic, Azienda Ospedaliero-Universitaria delle Marche, Ancona, Italy; 9 Department of Neuroscience, Reproductive Science and Odontostomatology, University of Naples Federico II, Naples, Italy; 10 Department of Biomedical Sciences, Division of Physiology, University of Cagliari, Monserrato, Cagliari, Italy; 11 Dipartimento Di Psicologia E Scienze Della Salute, Centro Direzionale, Università Telematica Pegaso, Naples, Italy; 12 Diabetes Unit, “S. Camillo-Forlanini” Hospital, Rome, Italy; 13 Centro Specialistico di Dietetica e Nutrizione Clinica Matrik, Bologna, Italy; 14 Azienda Ospedaliera San Camillo Forlanini, Rome, Italy

**Correction: Current Nutrition Reports (2026) 15:70**

**https://doi.org/10.1007/s13668-026-00790-0**

In the original version of this article, Odette Misa Sonia Hassan, Annalisa Maghetti, and Maria Grazia Carbonelli were omitted from the author list. They should be included as the 12th, 13th, and 14th authors.

The original article has been corrected.

